# Low-Cost Ethanol Concentration Sensor Based on a Balloon-like Curved Optical Fiber in a Mach–Zehnder Interferometric Configuration

**DOI:** 10.3390/s26123740

**Published:** 2026-06-12

**Authors:** Luis F. Espejo-Bayona, Sindi D. Horta-Piñeres, Duber A. Avila

**Affiliations:** Laboratorio de Óptica e Informática, Departamento de Física, Química y Afines, Facultad de Ciencias Básicas, Universidad Popular del Cesar, Valledupar 200001, Cesar, Colombia; shorta@unicesar.edu.co (S.D.H.-P.); duberavilap@unicesar.edu.co (D.A.A.)

**Keywords:** interferometric, curved optical fibers, Mach–Zehnder interferometric, ethanol concentration sensor, curved optical fiber balloon-like

## Abstract

We report an optical configuration to measure the concentration of ethanol (EtOH) using a light-guiding mechanism based on optical fibers in a Mach–Zehnder-type interferometric configuration. The proposed configuration takes advantage of the high sensitivity of fiber optic interferometers, allowing variations in EtOH concentration to be detected from shifts in the interference patterns. The interferometric configuration was implemented using a single-mode optical fiber curved into a balloon shape. During the experiment, some spectral stability and hysteresis tests were performed on the device, obtaining good results. During the characterization stage for measuring EtOH concentration in the 40–71% *v*/*v* range, performance parameters were evaluated, achieving EtOH concentration measurements in the 1500–1600 nm wavelength range. The sensitivity of the devices was evaluated in terms of the geometric parameters of the interferometric curved optical fibers, obtaining a maximum sensitivity of 0.1174 nm/% *v*/*v*. In this case, the transmitted optical signal analyzed at the end of the optical fiber allowed the identification of spectral shifts toward longer wavelengths with increasing EtOH concentration. In the study, the behavior of the spectral parameters of the interferometric curved optical fibers was analyzed as a function of the geometric parameters, highlighting their low cost, easy tuning control of minimum intensity peaks and good sensitivity.

## 1. Introduction

Optical interferometry is considered a measurement technique based on the superposition of light waves, which allows the detection of minimal variations in the phase, amplitude, or frequency of a light beam. These devices, in their various configurations, can offer high sensitivity and resolution for measuring different variables in various scientific fields, such as engineering, medicine, biology, environmental monitoring, and physical and chemical sciences, among others. In particular, their ability to measure spatial displacements at the nanometer scale and detect refractive index gradients makes them a valuable tool for the development of high-precision optical sensors. In recent years, different interferometric configurations have been explored, achieving significant progress in the development of new interferometric configurations, among which we can mention: free-space interferometers, chip-based interferometers, and fiber-based interferometers [1]. In the case of interferometers based on optical fibers, the most studied configurations in the sensing field are Mach–Zehnder (MZI) [2], Michelson [3], and Fabry–Perot interferometers [4]. In the specific case of MZI interferometers, these are notable for their high sensitivity to changes in refractive index, making them ideal for applications in the fields of chemistry and biology. Michelson interferometers offer a compact and stable configuration, while Fabry–Perot interferometers enable high spectral resolution measurements. These configurations have been widely used in the detection and measurement of substances such as ethanol, methanol, and other volatile compounds, demonstrating their versatility in different measurement environments. However, challenges related to miniaturization, robustness, and adaptability to real-world conditions persist, motivating the exploration of new interferometric designs that offer improved performance. In conclusion, interferometric systems based on optical fibers constitute a solution that allows miniaturization, robustness, flexibility, high spectral resolution and good sensitivity.

In general, according to the research reported by some authors, optical sensors based on optical fibers for the measurement of physical, chemical and biological variables have been reported in different configurations, where the most used configurations are optical fiber tapers [5,6], microstructured optical fibers [7], optical fibers by excitation of Whispering Gallery modes (WGMs) [8], photonic crystal optical fibers [9], Bragg optical fibers [10], long period grating optical fibers [11], coated optical fibers with excitation of surface optical plasmon resonances [12], and interferometric optical fibers [13], among others.

In this research, we propose the development of an interferometric configuration based on an interferometric curved optical fiber (ICOF) for the measurement of ethanol concentration in aqueous solutions. Measuring ethanol concentration in analytes is essential in various industrial, biomedical and environmental applications. In the food and beverage industry, monitoring ethanol concentration is necessary to ensure product quality, while in public health, alcohol detection is essential for metabolic monitoring and intoxication control. The developed device allowed in situ measurements of the EtOH concentration in an analyte with good sensitivity and reproducibility. The objective is to report the sensitivity of an optical sensing mechanism in a MZI configuration and evaluate its performance in terms of the wavelength shifts of the response signal. The results obtained provide valuable knowledge for the design of more efficient optical sensors, opening new possibilities for industrial and biomedical applications that require high-precision measurements in liquids.

## 2. Physical Principles and Operation

**Figure 1:**
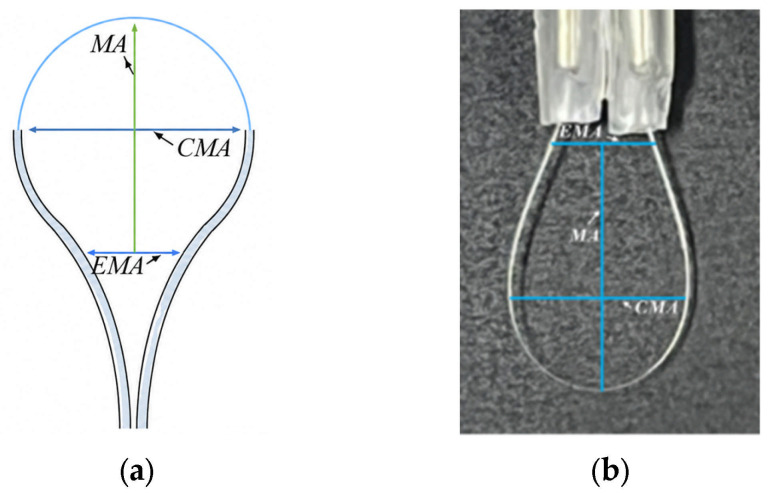
Mach–Zehnder-type sensor configurations. (**a**) General schematic of the structure, where MA denotes the major axial axis, CMA the central minor axial axis, and EMA the external minor axial axis, and (**b**) photograph of the fabricated device.

Among the different configurations of optical sensors based on optical fibers, MZIs have been shown to be especially effective in detecting small changes in the refractive index of the surrounding medium [14]. These characteristics make them ideal candidates for measuring ethanol concentration in aqueous media, since variations in the composition of a solution directly affect its refractive index, generating shifts in the interference patterns [15,16]. Previous research in the field of optical sensing, reported by different authors, has explored various optical fiber configurations for physical and chemical sensing applications, but there are challenges related to optimizing sensitivity and stability. In this research, we propose a measuring device based on an ICOF in a Mach–Zehnder configuration that has the shape of a balloon [17,18]. The proposed configuration would allow for adjustment of the sensing length, fiber curvature level, spectral position of the intensity minima, and the number of peaks, improving its robustness and flexibility compared to other optical fiber configurations. Figure 1a illustrates the general schematic of the device, which exhibits a balloon-like geometry with an approximately constant radius. Figure 1b shows an image of the configuration fabricated in the laboratory, where MA is the major axial axis, CMA is the central minor axial axis, and EMA is the external minor axial axis.

Fiber optic sensors in MZI configuration base their operating principle on the superposition between two coherent light waves, resulting in the generation of an interference pattern whose intensity depends on the phase difference between the waves. Generally, in configurations where waveguides are used as a light-guiding mechanism, interference can originate through two mechanisms: First, through the superposition between the guided modes of the core and the modes of the cladding in an optical fiber, and the second mechanism, through the division of light into two arms that travel along different optical paths before the optical signal recombination process. The intensity and power transmitted in optical fiber interferometers used for optical sensing experience the same physical principles of operation as robust interferometers, where interference fringes reveal deep peaks of minimum intensity in the spectrum, which can shift depending on the behavior of the external medium.

### 2.1. Mach–Zehnder Interferometers Based on Optical Waveguides

**Figure 2:**
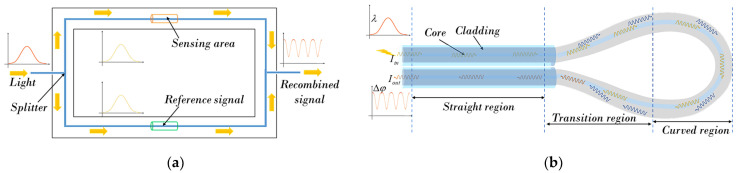
General schematic of two types of Mach–Zehnder interferometers according to their geometric configuration. (**a**) MZI interferometer with separate physical arms and (**b**) Mach–Zehnder interferometer based on curved optical fibers.

Optical sensors based on optical fibers in a MZI configuration can be analyzed using the mathematical model of a classic MZ interferometer. In this scheme, an incident light beam is split by a beam splitter, generating two beams that travel along independent optical arms. These beams are then recombined through a coupler, producing an interference pattern whose intensity depends on the accumulated phase difference between both paths, as illustrated in Figure 2a. When the interferometer is used for optical sensing, a small area of one of the optical arms is selected and functionalized as a sensitive region, where variations in the optical path due to changes in the physical length of the arm or changes in the properties of the surrounding medium can alter the phase of the propagated light, causing displacements of the interference pattern. On the other hand, in the case of MZI sensors based on curved optical fibers, light is coupled to the core of a straight section of a single-mode optical fiber, causing the excitation of the fundamental mode inside the optical fiber.

The light then passes through the transition region, where a fraction of the light is irradiated from the core to the cladding under the guiding condition, and then reaches the curved region (sensing zone). In this latter region, there is multimodal coupling, where the core modes give rise to higher-order modes in the cladding. In this case, the light guided by the core behaves as a reference signal, while the other part of the light that propagates between the cladding and the external medium behaves as a modulable signal. In the sensing zone, the light propagated at the cladding-external medium interface interacts with the medium and/or analyte and subsequently recombines with the reference signal, forming a characteristic interference spectrum. The schemes presented in Figure 2a,b are functionally equivalent; however, the curved optical fiber-based configuration should be considered physically as an asymmetric MZ interferometer, since the geometric differences between the core (typically ~9 μm) and cladding (usually ~125 μm) diameters are relatively large, resulting in modal coupling from the core to the cladding with different optical path lengths. This structural asymmetry leads to: (i) a significant difference in the lengths of the interferometric arms, and (ii) unequal modal coupling conditions between the core and cladding. These distinctive features could directly affect the device’s sensitivity.

The Mach–Zehnder interferometric configurations based on curved optical fibers proposed in this research allow the generation of modal interference fringes, observable in the transmission spectrum as peaks of minimum intensity at specific wavelengths. This spectral behavior is analogous to that reported in other widely studied optical platforms for optical sensing, such as long-period fiber gratings (LPFGs), fiber Bragg gratings (FBGs), tilted Fiber Bragg Gratings (TFBGs), and metal-coated functionalized optical fibers operating by surface plasmon resonance (SPR) or localized surface plasmon resonance (LSPR). However, compared to these configurations, the curved optical fiber balloon-like employed in this research offers distinct advantages in terms of structural simplicity, fabrication cost, and versatility for peak tuning. First, its implementation does not require complex microstructuring processes, inscription of Bragg gratings in optical fibers, metallic coatings, or surface functionalization to induce intense spectral valleys, significantly reducing cost and experimental complexity. Second, the position, depth, and response of the minimum intensity peaks can be tuned by adjusting geometric parameters of the structure, such as the radius of curvature, major axial length, minor axial lengths, and the characteristic dimensions of the deformed section. This ease of geometric tuning is a significant advantage over configurations such as LPFG, FBG, SPR, and LSPR, in which the appearance and location of intense valleys are caused by the periodic perturbation of the fiber’s refractive index (as in LPFGs and FBGs), the deposition of films on the fiber surface (SPR) and/or the deposition of metallic nanoparticles on the surface (LSPR). Achieving these effects in optical fibers generally requires specialized inscription or functionalization techniques, which make their fabrication more complex and typically more expensive than the configuration proposed in this study. Therefore, the selected configuration combines a relatively simple fabrication architecture with a well-defined and configurable spectral response, making it a promising alternative for developing low-cost, high-sensitivity, and reproducible optical sensors.

### 2.2. Results of Mathematical Modeling and Simulation of the MZI Configuration Based on Curved Optical Fibers

The experimental configuration shown in Figure 2b, whose photograph is presented in Figure 1b, was implemented using a standard Corning SMF-28 single-mode optical fiber with a core diameter of 8.2 μm and a cladding diameter of approximately 125 μm. The fiber curvature can be mechanically induced, and variable geometric parameters can be guaranteed to form the balloon-like structure. The geometric parameters of the configuration, such as the outer length of the minor axis, the central length of the minor axis, and the major axis length, can be modified and tuned to control the transmission response of the device in terms of the number of peaks and the tuning of the minimum intensity peaks. The curved optical fiber configuration exhibits a behavior analogous to an asymmetric Mach–Zehnder interferometric configuration, where the arms of the interferometer correspond to the effective lengths traveled by the light in the core $L_{eff,cor}$ and the cladding $L_{cla,cor}$ [19,20]. Generally, for an asymmetric MZI of optical fibers, light is coupled into the straight region of the fiber input port from a light source, exciting the fundamental mode in the core. Subsequently, the light reaches the transition region (low curvature zone), and a division of the light intensity occurs, where a small fraction propagates as modes in the core $t_{a}$, while the other fraction of the divided light propagates as modes in the cladding $1-t_{a}$, $t_{a}$ where the power division ratio is. Subsequently, the light reaches the curved region (high curvature zone), causing part of the cladding modes to leak into the external medium as an evanescent field. In this case, the cladding modes shift toward the exterior, increasing the evanescent field in the area and allowing direct interaction of the light with the analyte. Finally, the light recombines at the fiber’s output port, resulting in well-defined transmission spectra. For appropriate mathematical modeling, we can assume a superposition between the propagated modes in the nucleus with an effective refractive index $n_{eff,cor}$ and the propagated modes in the cladding with an effective refractive index $n_{eff,cla}$. In the curved region (sensing zone), the interaction length of each arm suffers losses quantified through the absorption coefficient of the core $\alpha_{cor}$ and the absorption coefficient of the $\alpha_{cla}$, respectively. The phase accumulation is related to the propagation constants in each of the interferometric arms $\beta_{cor}$ and $\beta_{cla}$.

(1) $$\Delta \varphi (\lambda ,n_{ext})=\varphi_{cor}(\lambda )-\varphi_{cla}(\lambda ,n_{ext})=\beta_{cor}(\lambda )L_{eff,cor}-\beta_{cla}(\lambda ,n_{ext})L_{eff,cla}\;\;\;\;\;\;\;\;\;$$

where $n_{ext}$ represents the refractive index of the external medium (a mixture of water and ethanol). The propagation constants can be expressed in terms of the effective refractive index through mathematical expressions [21,22]:

(2) $$\begin{array}{c} \beta_{cla}(\lambda ,n_{ext})=\frac{2\pi }{\lambda }n_{eff,cla}(\lambda ,n_{ext})\;\\ \beta_{cor}(\lambda )=\frac{2\pi }{\lambda }n_{eff,cor}(\lambda ) \end{array}\;\;\;\;\;\;\;\;$$

where the effective refractive indices exhibit dispersive behavior

(3) $$\begin{array}{c} n_{eff,cor}=n_{eff,cor}(\lambda )\\ n_{eff,cla}=n_{eff,cla}(\lambda ) \end{array}\;\;\;\;\;\;\;\;\;\;$$

The light intensity resulting from the superposition $I_{out}$ collected at the fiber end is determined by the following expression [21]:

(4) $$I_{out}=t_{a}I_{i}e^{\left(-\alpha_{cor}L_{eff,cor}\right)}+(1-t_{a})I_{i}e^{\left(-\alpha_{cla}L_{eff,cla}\right)}+2\sqrt{t_{a}(1-t_{a})}I_{i}e^{-\frac{\left(\alpha_{cor}L_{eff,cor}+\alpha_{cla}L_{eff,cla}\right)}{2}}Cos\Delta \varphi \;\;\;\;\;\;\;\;\;\;$$

where $I_{i}$ is the incident intensity, and Δφ represents the phase difference given by Equation (1) [20].

On the other hand, taking into account that the variations in ethanol concentration in binary liquid solutions have been addressed by some authors, it is possible to correlate the concentrations of ethanol in water with the refractive indices of the substance $n_{ext}$ through different models studied in the literature [23,24]. In this way, we can build a mathematical model initially considering differences between the effective lengths in each of the interferometer arms, where the transmission minima can be determined from the destructive interference condition from two consecutive minima [22,25].

(5) $$\Delta \varphi (\lambda ,n_{ext})=(2m+1)\pi \;\;\;\;\;\;\;\;\;\;$$

where m ∈ Z is a modal constant. By making the relevant substitutions, we obtain the condition for minimum intensity peaks as a function of the indices and effective lengths for different modes [21].

(6) $$(\frac{2\pi }{\lambda })\left[n_{eff,cor}(\lambda_{m})L_{eff,cor}-n_{eff,cla}(\lambda_{m},n_{ext})L_{eff,cla}\right]=(2m+1)\pi \;\;\;\;\;\;\;\;\;\;$$

Considering that the sensitivity of the device in the experiment is determined from the rate of variations in the wavelengths of minimum intensity in terms of the concentration $S_{c}=\Delta \lambda_{\min}/\Delta c$, mathematically, it can be implicitly determined by the expression:

(7) $$S_{c}\equiv \frac{d\lambda_{\min}}{dc}=\frac{d\lambda_{\min}}{dn_{ext}}\cdot \frac{dn_{ext}}{dc}\;\;\;\;\;\;\;\;\;$$

On the other hand, to determine the derivative of the wavelength in terms of the external refractive index, we implicitly derive Equation (5):

(8) $$\frac{d\lambda }{dn_{ext}}=-\frac{\frac{\partial \Delta \varphi }{\partial n_{ext}}}{\frac{\partial \Delta \varphi }{\partial \lambda }}\;\;\;\;\;\;\;\;\;$$

In this case, the partial derivatives required in Equation (8) are obtained from expressions (1) and (2).

(9) $$\frac{\partial \Delta \varphi }{\partial n_{ext}}=-\frac{2\pi }{\lambda }L_{eff,cla}\frac{\partial n_{eff,cla}}{\partial n_{ext}}\;\;\;\;\;\;\;\;\;\;$$

(10) $$\frac{\partial \Delta \varphi (\lambda ,n_{ext})}{\partial \lambda }=-\frac{2\pi }{\lambda^{2}}\left[n_{eff,cor}L_{eff,cor}-n_{eff,cla}L_{eff,cla}\right]+\frac{2\pi }{\lambda }\left[L_{eff,cor}\frac{\partial n_{eff,cor}}{\partial \lambda }-L_{eff,cla}\frac{\partial n_{eff,cla}}{\partial \lambda }\right]\;\;\;\;\;\;\;\;\;\;$$

By omitting dispersive effects $\partial n_{eff}/\partial \lambda \approx 0$, simplifying using expression (6), and finally replacing in expression (8), we have [26].

(11) $$\frac{d\lambda }{dn_{ext}}\approx \frac{2L_{eff,cla}}{\left(2m+1\right)}\frac{\partial n_{eff,cla}}{\partial n_{ext}}$$

To evaluate the variation of the external refractive index in Equation (7) with respect to the EtOH concentration, it is possible to use an approximation using the simple Gladstone–Dale model to relate the refractive index of the external medium to the EtOH concentration in water. In this case, in a heterogeneous mixture composed of EtOH and water, the refractive index exhibits a linear behavior with the concentration [27,28]. In this way, the derivative of the refractive index of the EtOH–water mixture with respect to concentration is a constant, and when included in Equation (7), does not change the sensitivity behavior. The final sensitivity could be expressed proportionally using the equation:

(12) $$S_{c}∼\frac{2L_{eff,cla}}{\left(2m+1\right)}\frac{\partial n_{eff,cla}}{\partial n_{ext}}\;\;\;\;\;\;\;\;\;$$

The final equation for sensitivity demonstrates the importance of determining the effective cladding length and the refractive optical responsivity of the mode in the cladding due to changes in the external refractive index because of EtOH variations. The final expression for sensitivity reveals a behavior dependent on the cladding characteristics. In this case, the sensitivity depends linearly on the cladding length, which could be obtained numerically from the integration of the local effective index averaged by the modal power distribution in the cladding modes. On the other hand, sensitivity directly depends on the variations in the cladding’s refractive index relative to changes in the external refractive index, indicating that the greater the evanescent field in the sensing zone, the greater the device’s optical responsivity. This latter term could be obtained using the coupled mode theory (CMT) complemented by numerical techniques.

**Figure 3:**
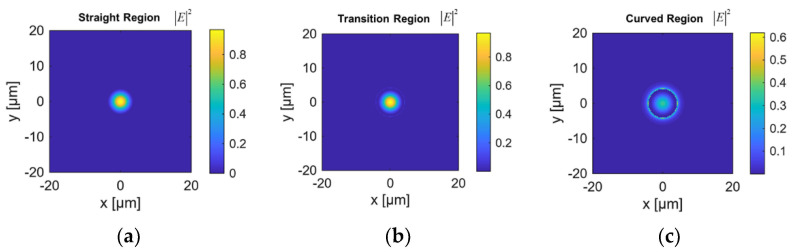
Contour maps of modal intensity in different regions: (**a**) straight region, (**b**) transition region and (**c**) curved region.

According to the simulation, Figure 3 shows contour maps of the modal intensity in the different regions of the configuration. Figure 3a shows the fundamental mode $LP_{01}$ in the core of the optical fiber in the straight region of the configuration. In this case, a field is strongly confined within the radius of the core, which serves as a reference as a guided field with minimal losses and a limited evanescent field in its vicinity.

In the transition region, the core modes begin to couple to the cladding modes, and the transmitted energy begins to redistribute, as observed in Figure 3b. In this case, the core modes begin to deform, and a stronger evanescent field is observed in the cladding. The simulation shows a small concentric ring around the main mode. Finally, in the curved region, a more intense concentric ring is observed compared to the transition region, demonstrating greater field deformation, which indicates an increase in the evanescent field fraction, as observed in Figure 3c.

**Figure 4:**
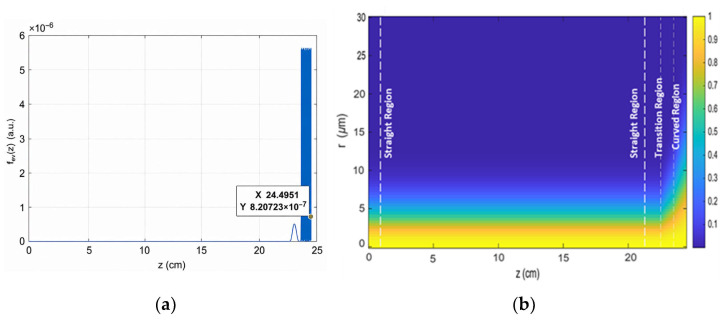
Longitudinally distributed power fraction: (**a**) longitudinal fraction of evanescent field in a 1D plot and (**b**) contour map of the evanescent field fraction in a contour plot for the straight, transition and curved regions.

On the other hand, regarding the behavior of the power fraction in each of the regions, it was possible to obtain a contour plot of the longitudinal power distribution along the three regions. Figure 4a shows the behavior of the optical field fraction in the three regions for specific lengths. In the straight region of 22.5 cm length, the power remains in the core (mode), with no energy transfer observed towards the cladding or the external medium. In the transition region between 22.5 and 23.5 μm, a moderate increase in power is detailed in this zone, indicating a significant excitation of the cladding modes, and finally, in the curved region between 23.5 and 24.5 μm, the fraction of the optical power increases significantly due to radiative losses, observing a significant evanescent field in this zone. The results show that the contribution of the evanescent field comes mainly from the excited modes of the cladding, because the core modes are still strongly confined. In the curved region, the cladding’s optical field penetrates the surrounding medium, maximizing the interaction of light with the analyte. Furthermore, Figure 4b shows a longitudinal contour map that relates the fraction of the evanescent field along the longitudinal distance. In this case, the map reveals that when traversing the configuration from the straight region to the transition region, an increase in the intensity of the optical field is observed at greater longitudinal distances, resulting in an energy intensification effect, observed in the increase in yellow color in the color bar diagram. The map indicates that the greatest light intensity occurs in the curved region, where a significant increase in optical intensity levels is observed.

### 2.3. Manufacturing and Design of the Device

In the development of this research, an experimental configuration for the measurement of EtOH concentration was characterized based on a curved optical fiber in an MZ-type interferometric configuration, which was exposed to changes in EtOH concentration from an analyte with a binary mixture of ethanol and water. The reported configuration was fabricated from a small, approximately 60 cm, uncoated SMF28 single-mode optical fiber, along a length of approximately 10 mm, which was manually curved. Each end of the curved fiber was secured in commercial optical-fiber protection sleeves to improve the stability of the device’s response. The end points of the curved fiber were then joined to an FC pathcord via a fiber optic splicer. During the experiment, it was possible to modify the geometric parameters of the transition region and the curvature region, successfully modifying the device’s transmission response. The final device configuration is detailed in Figure 1b.

## 3. Results

The designed and fabricated interferometric setup was evaluated to quantify the ethanol concentration in a binary liquid solution using EtOH and deionized water as solvents. To characterize the performance of the device, its optical response was measured with analytes of different EtOH concentrations varying in the range of 40% *v*/*v* to 71% *v*/*v*. In both configurations, the results demonstrated a correlation between the shifts of the minimum intensity peaks and the measured concentrations, evidencing sensitivity.

**Figure 5:**
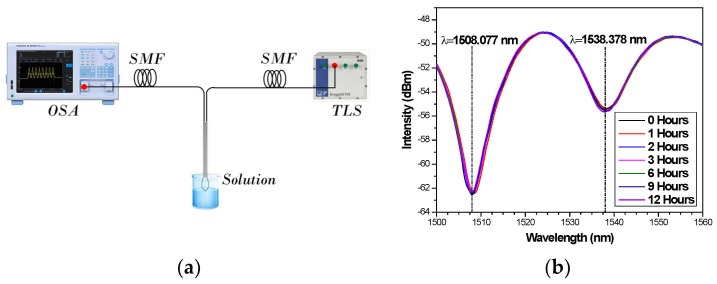
(**a**) Experimental setup for ethanol concentration measurement using the balloon-like curved optical fiber configuration and (**b**) temporal stability analysis of the balloon-like curved optical fiber configuration.

To characterize the performance of the proposed configuration for measuring EtOH concentration, the experimental setup shown in Figure 5 was developed. The system employs a tunable laser light source (TLS, Braggmeter FS 22DI) with an operating spectral range of 1500 to 1600 nm, and an optical spectrum analyzer (OSA, Yokogawa AQ6370E) was used as an interrogator. The light coming from the TLS is coupled to the core of the optical fiber, and subsequently, the light reaches the transition zone, where a modal coupling begins to be induced from the core modes to the cladding modes. The light then reaches the curvature region, where a fraction of the light is confined in the core (core modes), while the other fraction is confined in the cladding (cladding modes). On the other hand, in the central region of curvature (sensing zone), the cladding modes penetrate the external medium as an evanescent field. Once the light passes through the sensing zone, the core and cladding modes recombine, generating a pattern with minimum intensity peaks that are recorded by the OSA. For characterization, the measuring device is immersed in an aqueous solution with different concentrations of EtOH.

The transmission spectra of the balloon-like curved optical fiber configuration exhibit excellent temporal stability. Figure 5b shows the transmission spectra over sufficiently long periods (0–12 h), where two peaks of minimum intensity are evident.

**Figure 6:**
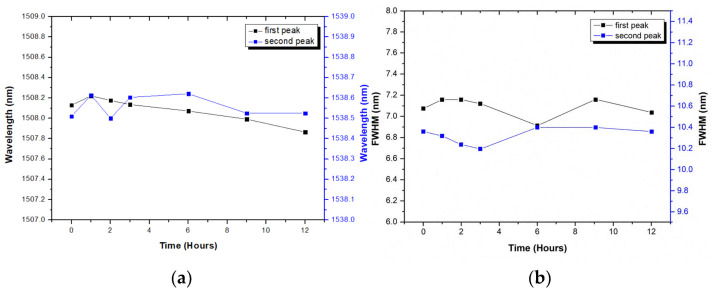
Stability analysis over time: (**a**) behavior of the central wavelength of the first and second peaks and (**b**) behavior of the FWHM of the first and second peaks.

Figure 6a shows a graph relating to the small spectral shifts of the minimum intensity peaks over time. The results reveal that, during the twelve hours of measurement, the spectral position of the first peak varied from 1507.9 nm to 1508.2 nm, as observed in the black line, and for the second peak, the spectral position varied between 1538.4 nm and 1538.7 nm, as observed in the blue line. On the other hand, Figure 6b shows the behavior of the Full Width at Half Maximum (FWHM) over time, where changes in the FWHM are evident in the range of 6.92 nm to 7.16 nm corresponding to the first peak, as detailed in the black curve. For the second peak, the FWHM varied from 6.93 nm to 7.17 nm, as detailed in the blue curve. The analysis of the device stability shows that, during a 12 h measurement period, the wavelength of the minimum intensity peaks showed a maximum deviation of approximately 0.3 nm for both peaks, while the FWHM experienced a variation of 0.24 nm for both peaks. These low-magnitude fluctuations indicate that the MZ interferometric setup based on curved fibers maintains a stable response under controlled experimental conditions.

During the experiment, it was possible to obtain different interferometric curved optical fibers (ICOF) with different geometric parameters, as detailed in Table 1 with the parameters schematized in Figure 1b.

**Figure 7:**
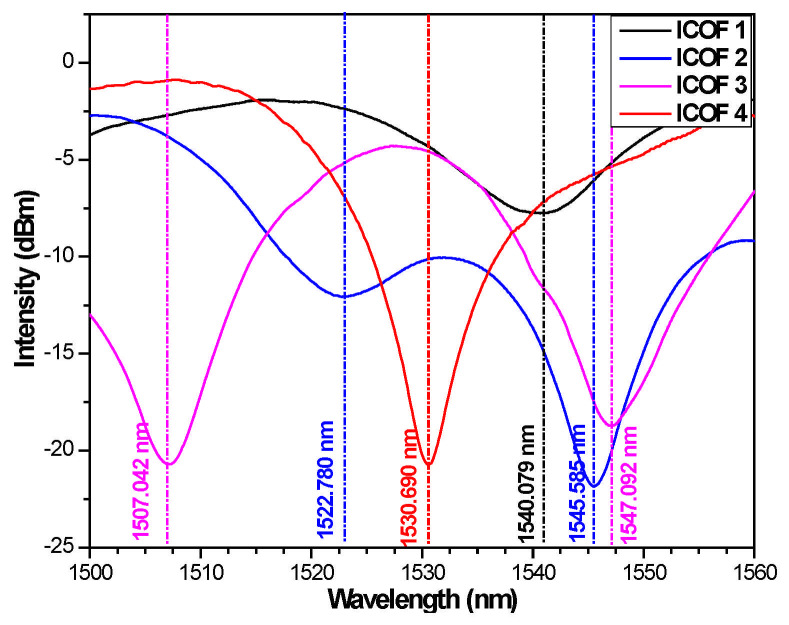
Transmission spectra of the different ICOFs with the geometric parameters defined in Table 1.

Figure 7 shows the behavior of the optical response transmitted by the different ICOF samples in the range of 1500–1560 nm, observing different spectral responses, depending on the specific geometric parameters of each ICOF.

According to the comparative results of the spectral response of the device as a function of the geometric parameters of the ICOF, the response shows a significant dependence on the length of the major axis *MA*. According to the results observed in the experiment, the device experiences different behaviors depending on the number of minima detected in the specific evaluation range. In this case, the device experiences two different modes of operation: (i) a single-minimum mode and (ii) a two-minimum mode of operation. Using an experimental data fitting technique, it was possible to find relationships that allow determining the dependence of the spectral parameters on the geometric parameters. To determine the position of the minimum intensity peaks in terms of the major axis length *MA*, in the case of the single minimum mode of operation, we can use a linear fit of the form $\lambda \left[nm\right]=a+bMA$ for MA≥15mm, and for the case of the two minimum modes of operation, we use a fit for each of the minimum intensity peaks of the form: $\lambda_{1}\left[nm\right]\approx c+dMA$ and $\lambda_{2}\left[nm\right]\approx e+fMA$, being a, b, c, d, e and f arbitrary constants obtained by the experimental data fitting technique: a=1398.84, b=8.56, c=1379.85, d=9.97, e=1559.27 and f=0.96. For the Free Spectral Range (FSR) case, the linear fit is of the form $FSR\left[nm\right]\approx g+hMA$ with constants g=179.42 and h=10.93. Regarding the FWHM, for the case of the single minimum operating mode, the adjustment expression is $FWHM_{P1}\left[nm\right]\approx i+kMA$, and for the two-minimum mode of operation for each peak, we have: $FWHM_{P1}\left[nm\right]\approx l+mMA$ and $FWHM_{P2}\left[nm\right]\approx n+oMA$ with constants: i=−73.90, k=5.39, l=53.91, m=3.30, n=60.76 and o=3.73.

From the analysis of this experiment, we can reveal that a reduction in the MA causes the device to evolve from the single minimum mode of operation $\left(MA\approx 16.5-15.0\;mm\right)$ to the two-minimum mode of operation $\left(MA\approx 14.3-12.8\;mm\right)$. On the other hand, in relation to the separation between FSR peaks, this parameter increases from ∼22.8 nm to ∼40.1 nm by decreasing the *MA* of 14.33 mm to 12.75 mm, according to the corresponding adjustment equation. The quality factor indicator FWHM is reduced from ∼15 nm to ∼8 nm when the *MA* decreases (single minimum mode of operation) but increases from ∼7 nm to ∼13 nm when the curvature increases (two-minimum mode of operation). In this way, *MA* governs the mode of operation (number of minima) and the spectral sensitivity, while *EMA* and *CMA* modulate the specific curvature.

**Figure 8:**
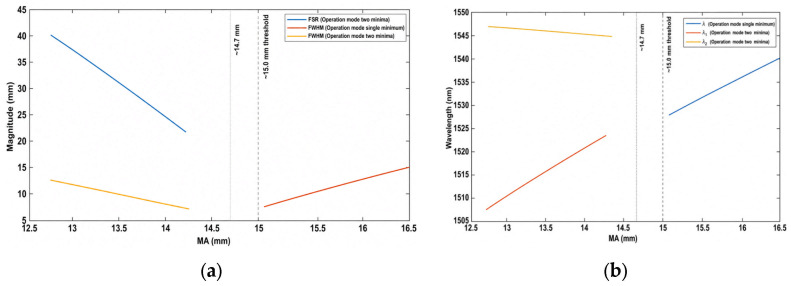
Relationship of spectral parameters and geometric parameters: (**a**) design parameters that relate FSR and FWHM with MA and (**b**) design parameters that relate minimum positions with MA.

For a detailed analysis of the device geometry on the behavior of the spectral parameters, it is possible to conclude that the overall curvature of the balloon-like curved optical fiber is controlled by *MA*, which is evident in Figure 8a,b. In this case, by decreasing *MA,* the radius of curvature in the sensing region is reduced, causing: (i) a displacement of the modal field from the core to the external medium, allowing the coupling of the core modes to the cladding, and (ii) modification of the effective refraction indices due to curvature losses, modal dispersion and the photoelastic effect. These effects manage to reduce the product between the difference of the effective refractive index and the effective length, managing to increase the FSR, which is in agreement with the experimental results, since when *MA* decreases, two intensity minima appear within the analysis range. According to Figure 8, during a smooth curvature (large *MA*), a single minimum is excited within the spectral window. On the other hand, increasing the curvature (smaller *MA*) and asymmetry (smaller *EMA/CMA*) excites a second peak of minimum intensity. According to empirical relationships, it is observed that by reducing *MA*, the minimum intensity peak with a shorter wavelength $\left(\lambda_{1}\right)$ shifts towards shorter wavelengths, while the minimum intensity peak with a longer wavelength shifts towards longer wavelengths. This can be explained by reducing the product of $\Delta n_{eff}L_{eff}$ with the curvature.

**Figure 9:**
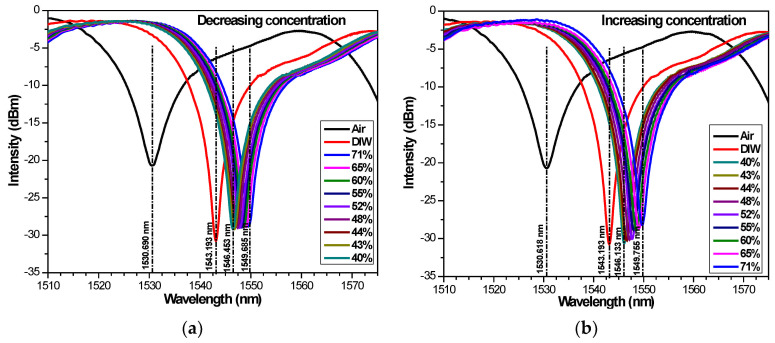
Characterization of the ICOF2 balloon-like interferometric curved fiber: (**a**) decreasing ethanol concentration and (**b**) increasing ethanol concentration.

Furthermore, a hysteresis analysis of the device response was performed. In this case, the behavior of the ICOF2 was evaluated with increasing and decreasing EtOH concentration gradients. The behavior of the displacement of the minimum intensity peaks as a function of the EtOH concentration is observed in Figure 9. In this case, Figure 9a reveals the device response behavior when the ethanol concentration is reduced, while Figure 9b reveals the device behavior when the EtOH concentration is increased. The experimental results reveal that the spectral positions of the interferometric peaks with the device suspended in the air are approximately in the same position, 1530.690 nm (decreasing concentration) and 1530.618 nm (increasing concentration). This same spectral stability behavior is evident when immersing the device in deionized water (DW), managing to maintain the spectral position at 1543.193 nm for increasing and decreasing EtOH concentrations. Figure 9a shows the position of the minimum intensity peak at 1549.685 nm for an ethanol concentration of 71% and 1546.453 for an ethanol concentration of 40%. This behavior shows that as the concentration of ethanol in the analyte decreases, there is a shift towards shorter wavelengths, while in Figure 9b, a peak of minimum intensity is observed at 1546.133 nm for a concentration of 40% and 1549.755 nm for a concentration of 71%, indicating that as the concentration of ethanol increases, there is a shift towards longer wavelengths.

**Figure 10:**
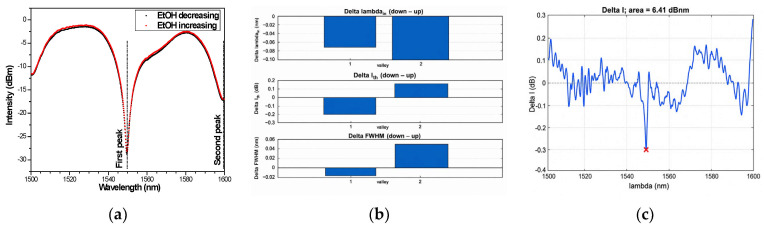
Hysteresis analysis of sample ICOF2 for 71% EtOH. (**a**) Comparison of concentration deviations at 71% EtOH with increasing and decreasing concentration, (**b**) graph of deviations during the scan, and (**c**) depth of intensity variations by wavelength.

In the hysteresis analysis, comparisons of the device response with ascending and descending EtOH concentration sweeps were developed; in this case, Figure 10a reveals the transmission response of the device at 71% EtOH concentration for ascending and descending concentration measurements. The spectral position of the minimum intensity peak is 1549.685 nm (decreasing concentration) and $\Delta \lambda =\lambda_{down}-\lambda_{up}$ (increasing concentration). The graph shows a second peak of minimum intensity, located at 1599.004 nm (decreasing concentration) and 1599.236 nm (increasing concentration).

Similarly, the behavior of intensity variations and FWHM was analyzed. In the upper part of Figure 10b, the behavior of the difference can be observed $\Delta \lambda =\lambda_{down}-\lambda_{up}$ according to the minimum intensity peaks for the 71% EtOH concentration. For the case of the first peak observed in Figure 10a, there was a spectral shift of −0.08 nm (first peak) and −0.12 nm (second peak). Regarding changes in intensity levels, Figure 10b (central figure) details a decrease of approximately −0.2 dBm (first peak) and an increase of approximately 0.2 dBm (second peak). Regarding the FWHM variations, a decrease of −0.02 nm (first peak) and an increase of approximately 0.05 nm were observed. Comparatively, it was found that the device (at a concentration of 71% EtOH) exhibited good stability in wavelength, intensity, and FWHM. On the other hand, it can be seen from Figure 10b (center) that the response of each minimum intensity peak can vary depending on the mode.

**Figure 11:**
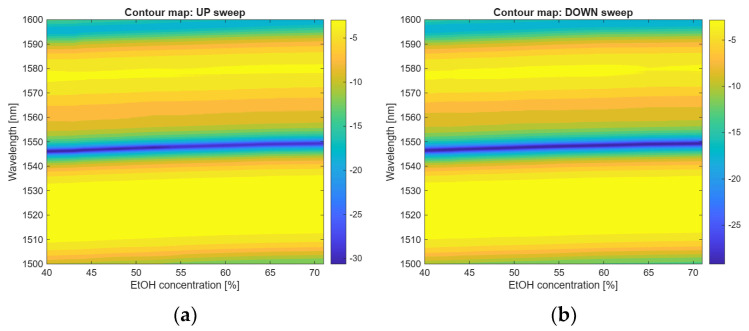
Contour maps of wavelength, concentration, and intensity behavior. (**a**) Concentration ramp-up test and (**b**) concentration ramp-down test.

Figure 10c reveals the intensity variations for each wavelength (1500–1600 nm). In this case, it is detailed that the variations in intensity are more evident in the positions of the minimum intensity peaks (approximately 1549 nm and 1599 nm). The area under the curve of the variations in wavelength is a parameter that can determine the deviation of the measurements (6.41 dBm*nm) during the sweep. In the contour map of Figure 11, it is possible to observe the response of the ICOF2 sample to the EtOH concentration for each wavelength, and its optical intensity levels. The contour map in Figure 11a reveals the behavior of the wavelength as a function of concentration for the intensity values in the ascent test, while Figure 11b reveals the same behavior for the descent test. The contour maps show a tilted linear zone with minimum intensity values (blue) around 1550 nm, which is related to the sensitivity of the device for the first minimum intensity peak.

**Figure 12:**
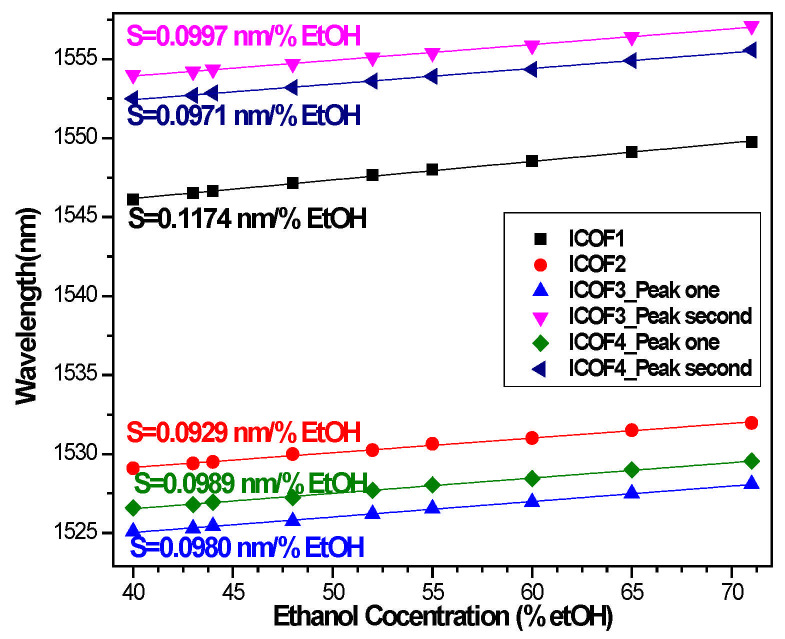
Sensitivity response for samples ICOF1, ICOF2, ICOF3 and ICOF4.

To evaluate the sensitivity level of the device, we analyzed the spectral shifts of the minimum intensity peaks as a function of changes in EtOH concentration. Once the level of displacement of the minimum intensity wavelength was analyzed for the different configurations developed (ICOF1, ICOF2, ICOF3 and ICOF4), a linear behavior pattern was evident for the sensitivity response. In this case, when evaluating the sensitivities of the different devices developed, we found that the best sensitivity was obtained with the ICOF1 sample (0.1174 nm/% EtOH). However, comparisons of the sensitivities of all the devices evaluated do not show significant differences. This behavior can be observed in Figure 12. In the case of the ICOF3 and ICOF4 devices, which present two peaks of minimum intensity, the experimental results suggest that the wavelength shift per unit concentration of EtOH is approximately equal.

A noteworthy aspect of this research is that various interferometric configurations-based balloon-shaped optical fibers have previously been reported in the literature. Such devices have been widely explored for the sensing of different physical and chemical variables, where the detection mechanism is mainly governed by spectral changes produced by variations in the surrounding refractive index. In this research, the results obtained differ from previous studies in both their purpose and implementation. Previous work has focused primarily on detecting temperature, humidity, displacement, or the simultaneous detection of refractive index and temperature using additional structural or functional elements such as waist-enlarged fusion taper, silica capillary sections, cascaded balloon-like bent fiber structures, and polymer/liquid coatings. Liu et al. improved the sensitivity for refractive index and temperature measurements by integrating a tapered-waist fiber (WEFT) into the balloon-shaped interferometer [22]. Ding et al. used a balloon-shaped fiber optic interferometer inside an ethanol-filled capillary to measure temperature through the thermo-optical response of ethanol [25]. Freitas et al. implemented a balloon-shaped sensor fabricated from a commercial single-mode optical fiber inside a silica capillary tube for the simultaneous detection of refractive index and temperature [17]. Tian et al. reported an interferometric sensor based on two cascaded balloon-shaped curved fibers to simultaneously measure micro-displacements and temperature. The overall complexity of the device was evaluated to achieve simultaneous measurements with good sensitivity [29]. In contrast, this research uses a single-mode curved optical fiber in a Mach–Zehnder interferometric configuration to directly measure ethanol concentration in the 40–71% *v*/*v* range. Table 2 summarizes a comparative analysis between the results reported in previous studies and those obtained in the present work. The proposed device operates without the need for additional coatings, surface functionalization, or specialized fabrication processes, thereby offering a simple and cost-effective sensing platform, and its spectral response can be tailored through the controlled adjustment of the geometric parameters. Furthermore, the incorporation of stability and hysteresis tests strengthens the practical relevance of the proposed sensor for liquid concentration monitoring.

## 4. Conclusions

During the development of the research, we implemented a simple, low-cost, and highly reproducible measurement mechanism based on a single-mode optical fiber curved in the shape of a balloon, which allows easy tuning of minimum intensity peaks according to the geometry of the configuration. The sensing mechanism uses the principle of optical interferometry with an analogy with MZ interferometers, allowing the superposition of the propagated modes in the core and the cladding, generating an interference pattern whose number of minimum intensity peaks depends on the geometry of the configuration. The research incorporated a theoretical model to explain the phenomenon, which allows the device’s sensitivity to be numerically determined based on the effective length of the cladding and the gradients of the effective refractive index of the cladding modes as a function of the external refractive index. The developed devices showed excellent stability of the transmission response over time, demonstrating good repeatability and reproducibility in the experiment. On the other hand, the simulations developed in the research reproduce the experimental results, providing a reliable measure of sensitivity. Regarding the device performance parameters, it was experimentally verified that the sensitivity for measuring the EtOH concentration presents a spectral modulation, whose sensitivity levels may depend on the position of the peak of minimum intensity analyzed, achieving a maximum sensitivity of 0.1174 nm/% *v*/*v* for a specific configuration. The developed configuration would allow the fabrication of low-cost optical sensing devices due to the relative ease of manufacturing the specific configuration, with sensitivity levels comparable to other measurement techniques. The device stands out for its simple design, control of minimum intensity peaks, good optical stability, and excellent sensitivity, with potential applications in various fields of science and engineering.

On the other hand, regarding the potential advantages of this configuration compared to other standard configurations, such as LPFG, FBG, TFBG, SPR/LSPR-based fibers, taper fibers, and other coated configurations, this device does not require periodic Bragg grating, metallic film deposition, nanoparticle coating, polymer functionalization, or the application of complex microstructuring processes to produce well-defined spectral minima with good sensitivity. In this case, the minimum intensity peaks are obtained through controlled modal coupling between the core and cladding modes, which are induced by the curvature of the balloon-shaped optical fiber. These advantages reduce manufacturing complexity and cost while preserving a stable, concentration-dependent spectral response. Thus, the position, number, depth, and linewidth of the minimum intensity peaks can be adjusted by modifying simple geometric parameters, such as the major axis, minor axes, and bend radius. These geometric tuning properties provide a degree of design flexibility compared to traditional configurations, where the spectral response is primarily determined by fixed periodic gratings or coatings deposited on the fiber surface. Therefore, the proposed configuration represents a simple, low-cost, reproducible, and easily adjustable alternative with potential applications in compact optical sensing platforms. Some of the specific applications of this device are in industry, particularly in the quality control of alcoholic beverages, live monitoring of ethanol–water mixtures and pharmaceuticals, verification of ethanol concentration in specific formulations, and chemical detection in laboratory tests. Furthermore, since the detection mechanism is based on variations in the refractive index induced by changes in the liquid’s composition, the configuration could be adapted for the detection of other binary mixtures of liquids or chemical analytes after proper calibration. Beyond measuring ethanol concentration, the described device represents a promising platform for the development of fiber optic biosensors, an area of significant interest over the past decade.

## Figures and Tables

**Table 1:** Geometric parameters of the ICOF.

Geometric Parameters				Spectral Response	
*Sample*	*External minor axis length EMA (mm)*	*Central minor axis length CMA (mm)*	*Major axis length MA (mm)*	*Spectral Position (nm)*	*FWHM (nm)*
ICOF1	5.746	11.423	16.495	1540.079	15.48
ICOF2	5.540	10.704	15.070	1530.690	9.04
ICOF3	5.481	10.167	14.331	Peak 1: 1522.780 Peak 2: 1545.585	Peak 1: 7.60 Peak 2: 7.64
ICOF4	5.488	9.378	12.753	Peak 1: 1507.042 Peak 2: 1547.092	Peak 1: 11.27 Peak 2: 13.24

**Table 2:** Comparative analysis of the characteristic aspects of this work and previously reported studies.

Ref.	Configuration	Measurement	Sensitivity	Main Limitation Relative to the Present Work	Advancement of the Present Work
[22]	Balloon-like interferometer with embedded WEFTs	RI, Temperature	340.9 nm/RIU; −0.162 nm/°C	Requires additional internal taper structure	Uses a simpler single curved SMF and is oriented to direct ethanol concentration sensing
[25]	Balloon-like SMF inside tube, filled with ethanol	Temperature	−1.145 nm/°C	Designed for temperature. Requires filling tube with ethanol	Measures ethanol concentration itself, no need for liquid-filled encapsulation architecture
[17]	Balloon-type sensor with silica capillary tube	RI, Temperature in acetic acid solutions	170.66 nm/RIU; −119.2 pm/°C	Composite capillary-based structure	Uses a single-fiber interferometric configuration and focuses on ethanol concentration in 40–71% *v*/*v*
[29]	Two cascaded balloon-like curved fibers	Displacement and Temperature	−318.8 pm/µm; 47.4 pm/°C	Requires two sensing sections, increasing complexity	Our structure is more compact and simpler, while still enabling concentration-dependent wavelength shifts
[18]	PVA-coated balloon-like SMF	Humidity	−1.927 nm/%RH	Requires functional polymer coating	Avoids added coatings and targets liquid ethanol concentration, with geometric tunability of spectral minima
Present work	SMF bent into a balloon-shaped MZI	Ethanol concentration	Max sensitivity 0.1174 nm/% *v*/*v*.	---	Direct ethanol concentration sensing, simple fabrication and geometric tunability

## Data Availability

Data underlying the results presented in this paper are available.
